# Occupational COVID-19 Exposures and Illnesses among Workers in California—Analysis of a New Occupational COVID-19 Surveillance System

**DOI:** 10.3390/ijerph20136307

**Published:** 2023-07-06

**Authors:** David Pham Bui, Kathryn Gibb, Martha Fiellin, Andrea Rodriguez, Claire Majka, Carolina Espineli, Elisabeth Gebreegziabher, Jennifer Flattery, Ximena P. Vergara

**Affiliations:** 1 Occupational Health Branch, California Department of Public Health, Richmond, CA 94804, USAmartha.fiellin@cdph.ca.gov (M.F.); andrea.rodriguez@cdph.ca.gov (A.R.); claire.majka@cdph.ca.gov (C.M.); carolina.espineli@cdph.ca.gov (C.E.); elisabeth.gebreegziabher@cdph.ca.gov (E.G.); jennifer.flattery@cdph.ca.gov (J.F.); ximena.vergara@cdph.ca.gov (X.P.V.); 2 Heluna Health, City of Industry, CA 91746, USA; 3 Public Health Institute, Oakland, CA 94607, USA

**Keywords:** COVID-19, occupational health, workers, surveillance, SARS-CoV-2

## Abstract

Little is known about occupational SARS-CoV-2 exposures and COVID-19 outcomes. We established a Doctor’s First Reports of Occupational Injury or Illness (DFR)-based surveillance system to study cases of work-related COVID-19 exposures and disease. The surveillance data included demographics, occupation, industry, exposure, and illness, details including hospitalization and lost work. We classified workers into ‘healthcare’, non-healthcare ‘public-facing’, or ‘other’ worker groups, and rural–urban commuting areas (RUCAs). We describe worker exposures and outcomes overall by worker group and RUCA. We analyzed 2848 COVID-19 DFRs representing workers in 22 detailed occupation groups and 19 industry groups. Most DFRs were for workers in metropolitan RUCAs (89%) and those in healthcare (42%) and public-facing (24%) worker groups. While DFRs were from 382 unique worksites, 52% were from four hospitals and one prison. Among 1063 DFRs with a suspected exposure, 73% suspected exposure to a patient or client. Few DFRs indicated hospitalization (3.9%); however, the proportion hospitalized was higher among nonmetropolitan (7.4%) and public-facing (6.7%) workers. While 56% of DFRs indicated some lost work time, the proportion was highest among public-facing (80%) workers. Healthcare and prison workers were the majority of reported occupational COVID-19 exposures and illnesses. The risk of COVID-19 hospitalization and lost work may be highest among nonmetropolitan and public-facing workers.

## 1. Introduction 

Early in the coronavirus disease 2019 (COVID-19) pandemic, outbreaks reported in healthcare, manufacturing, construction, and other industries highlighted the risk of COVID-19 to workers [1,2,3,4,5,6]. While stay-at-home orders were implemented to mitigate transmission, critical infrastructure workers continued to report to worksites where outbreaks and deaths ensued [6,7,8]. In California, while the full impact of COVID-19 among workers is not well understood, the potential burden is high—nearly three-quarters of confirmed COVID-19 cases and a third of deaths are among working-age individuals (18–64 years old); over 180,000 COVID-19 workers’ compensation claims have been filed; at least 12,000 workplace clusters have been identified involving more than 90,000 cases; and thousands of workers have died from COVID-19 [7,8,9]. Moreover, the pandemic has had social impacts on workers, including lost work, mental health stress, and other hardships which can further be exacerbated by post-COVID conditions [10,11,12,13]. 

Despite this burden of COVID-19 among workers, relatively little is known about work-related exposures and COVID-19. Infectious disease surveillance systems were unprepared to collect occupational data, thus presenting a barrier to assessing occupational COVID-19 risks [14,15]. Even in states that could collect case-patient industry and occupation, it remains unclear to what extent cases were due to workplace exposures [14]. California’s incidence of SARS-CoV-2 test positivity among workers by occupation and industry is undetermined, and signs and symptoms for worker populations have not yet been documented. Furthermore, while rural communities have experienced high COVID-19 mortality rates [16], faced barriers to tele-healthcare [17], and have less access to COVID-19 vaccines compared to urban counterparts [18], rural communities have largely been excluded from COVID-19 research, and little is known about their experience with occupational COVID-19 [19]. In order to guide workplace COVID-19 policies and recommendations, it is critical to understand which workers are exposed at work, how they may be exposed, and their health outcomes. 

In California, physicians are required to complete Doctor’s First Report of Occupational Injury or Illness (DFR) forms within five days of initial examination for work-related injuries or illnesses [20]. DFRs have been used by the California Department of Public Health (CDPH) since the 1940s for occupational injury and illness surveillance, including for pesticide exposures and work-related asthma [20,21]. We collected data from DFRs to develop a surveillance system for occupational SARS-CoV-2 exposures and COVID-19 [22]. This surveillance system was devised to systematically collect data on demographics, circumstances of exposures, use of personal protective equipment, and health outcomes. The objective of this study was to use DFR surveillance data to report on workers affected by occupational SARS-CoV-2 exposures and COVID-19, as well as their outcomes, including lost work time and hospitalizations. 

## 2. Materials and Methods

### 2.1. Study Design and Data Source

We analyzed data from the DFR surveillance system as of 14 November 2022 for this descriptive study. DFRs contain fields for patient demographics, work details (employer name, nature of business, and occupation), illness characteristics (exam, injury, or illness dates; circumstances of exposure and illness reported in patient and physician free-text narratives; subjective complaints; objective clinical findings; diagnoses; and treatment), and physician or treatment-facility information [23]. 

In January 2020, we began screening all received DFRs for COVID-19 diagnoses, relevant symptoms, or work-related COVID-19 exposures to identify all potential occupational COVID-19 exposures (i.e., working near, sharing the same work area, or close contact to a person with confirmed or suspected COVID-19) and cases (workers who test positive for SARS-CoV-2 or develop illness/symptoms consistent with SARS-CoV-2 infection from a suspected occupational exposure) (see Supplementary Materials for screening details). Trained staff abstracted all COVID-19-relevant DFRs into the surveillance database, using a standardized variable list to ensure accurate and consistent data abstraction. DFRs report discrete events of occupational SARS-CoV-2 exposures and/or COVID-19 and were the unit of analysis for this study; thus, a given worker could appear more than once in analyses if he/she had multiple discrete exposures or illnesses. All abstracted DFRs were analyzed, with the earliest exam date being 31 January 2020. Employer first reports (EFRs), a similar non-clinical mechanism for employers to report occupational injuries and illnesses to the state, are occasionally received and were included in analyses when relevant. EFRs typically contain less information about illnesses and outcomes than DFRs. 

### 2.2. Variables

#### 2.2.1. Demographic Variables

Worker demographics included age, sex, and rural–urban commuting area (RUCA). The worker’s residential zip code was used to determine the RUCA [24]. We used the 2010-RUCA zip-code file published by the US Department of Agriculture to assign workers to metropolitan (RUCA Codes 1–3) or nonmetropolitan (including RUCA Codes 4–6 for micropolitan, Codes 7–9 for small town, and Code 10 for rural area) designations. 

#### 2.2.2. Work-Related Variables

We used data from the employer’s nature of business field and employer name to code the employer’s industry to 4-digit 2012 Census Industry Codes. Worker occupation was manually coded to 2010 Census Occupation Codes (COC); both a detailed occupation and an occupation major group were assigned. Using the COCs, we classified workers using the Council of State and Territorial Epidemiologists Occupational Health Subcommittee Worker Group Assignment matrix [25]. This matrix classifies occupations into three groups based on potential occupational COVID-19 risk, including (1) healthcare workers likely to interact with COVID-19 patients, (2) public-facing workers who require routine face-to-face interaction with the public, and (3) all “other” workers who do not meet the criteria for healthcare or public-facing worker categories [25]. 

#### 2.2.3. Exposure-Related Variables

Exposure measures included the reported source of exposure, exposure site, and use of personal protective equipment (PPE). Abstractors used DFR narratives to ascertain a source of SARS-CoV-2 exposure (i.e., working near, sharing the same work area, or close contact to a person with confirmed or suspected COVID-19 while working). The source of exposure choices included patients, coworkers, clients or customers, personal (e.g., family member), unknown to the patient, or not stated in the DFR. DFRs could be coded to multiple exposure sources if indicated. Employer information and narratives were used to determine the patient’s exposure site; we grouped sites into several categories, including agriculture (e.g., farms), institutions (e.g., hospitals, prisons), manufacturing, other commercial facilities (e.g., retail), and other. We also attempted to abstract information from DFR narratives related to PPE use, including respirators, face shields or safety goggles, gloves, and gowns.

#### 2.2.4. Health Outcome Variables 

Outcomes include observed clinical signs and reported symptoms, hospitalizations, loss of work, and type of medical care sought. We abstracted mental health, respiratory, cardiovascular, nervous, dermatological, eye, gastrointestinal, renal, and constitutional/other signs and symptoms. We coded hospitalization as a binary outcome. Abstractors reviewed narratives, date last worked, and return-to-work fields to determine whether patients lost work time due to illness and isolation, or to quarantine related to an exposure. We assumed a Monday-through-Friday full-time work week for all patients and counted workdays between the date last worked and return-to-work date. In DFRs missing the date last worked, abstractors used a positive COVID-19 test date or symptom onset date as the starting date for lost time. When a return-to-work date was missing, the follow-up appointment date was used as the end date. We used provider and treatment facility information to determine the type of medical care sought (e.g., emergency room visit and telemedicine) to assess worker care-seeking behaviors.

### 2.3. Statistical Methods

We described DFRs by worker demographics, exposures, and outcomes, using descriptive statistics. We generated descriptive statistics for all variables and reported results for all workers, and then we further stratified this by RUCA, worker group assignment, and occupation major groups. Occupation denominators from the Current Population Survey (CPS), a household survey conducted by the US Census Bureau and Bureau of Labor Statistics, were used to estimate a reporting rate for all workers (defined as respondents who indicated that they were currently employed) *and* by occupation major groups in California [26]. The CPS Basic Monthly Survey data files were downloaded from the Integrated Public Use Microdata Series (IPUMS) website [27], and survey-weighted occupation population estimates were calculated with 95% confidence intervals (CIs). The CPS composite weight was used to replicate Bureau of Labor Statistics labor force estimates.

Reporting rates were calculated by dividing the number of DFRs for a given occupation group or RUCA designation by the occupation’s and RUCA designation’s population estimate and multiplying by 100,000 to obtain rates per 100,000 workers. We provide a total reporting rate based on average employment estimates for 2020 through 2021. Since a majority of DFRs were from 2020, we also report a 2020-only rate based on 2020 occupation estimates as a sensitivity analysis. All analyses were performed in R (R Core Team, Version 4.2.2, Vienna, Austria). 

## 3. Results

**Figure 1:**
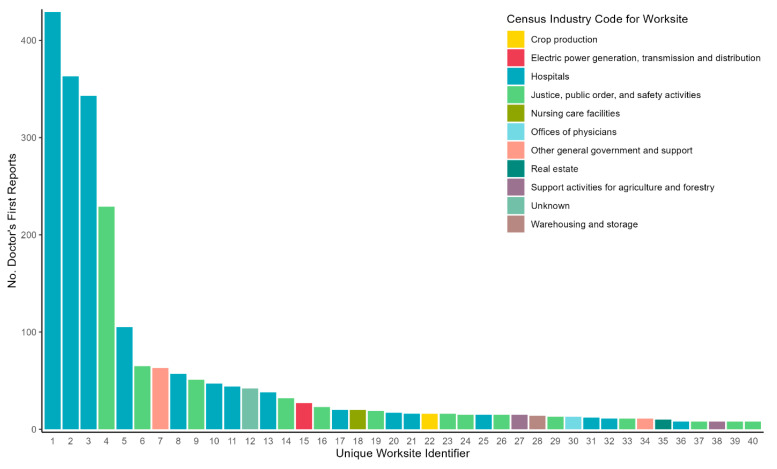
COVID-19-related Doctor’s First Report of Occupational Injury or Illness (DFR) frequency for 40 most represented unique worksites in California, January 2020–February 2022. Unique worksites by identifier are on the *x*-axis and colored by the census industry code that was coded for the worksite.

We analyzed 2848 COVID-19-related DFRs from 382 unique worksites, representing workers in 22 major occupation groups and 19 major industry groups (Table 1). Among the DFRs analyzed, 23 (<1%) were EFRs. Most DFRs listed exam dates in 2020 (n = 2035, 72%), followed by 2021 (n = 708, 25%) and 2022 (n = 81, 3%); 24 (<1%) were undated. About two-thirds of DFRs were generated for workers in healthcare (42%) and public-facing (24%) occupations, and 14% were classified as “other” workers. Notably, about a fifth (19%) of DFRs lacked occupation details. Major occupation groups with the most DFRs included healthcare practitioners and technical support (n = 914, 32%), protective services (n = 591, 21%), healthcare support (n = 242, 8.5%), and office and administrative support (n = 189, 7%). Over half (52%) of all DFRs were generated from just five worksites, including four hospitals (n = 1240, 44%) and one state prison (n = 229, 8%) (Figure 1).

### 3.1. Worker Demographics

The median age of workers represented in DFRs was 42 years (interquartile range: 32–51), and there were slightly more DFRs for female (51%) workers compared with male (45%) workers. Most DFRs were among workers residing in metropolitan (89%) RUCAs, and just 7% were from nonmetropolitan workers. When stratified by RUCA and worker group assignments, there was a greater proportion of healthcare workers in metropolitan (n = 1125, 44%) than in nonmetropolitan (n = 32, 17%) RUCAs (Table 1). The proportion of public-facing occupations was higher in nonmetropolitan (n = 86, 45.5%) compared to metropolitan (n = 566, 22%) RUCAs. 

### 3.2. Reporting Rates

**Figure 2:**
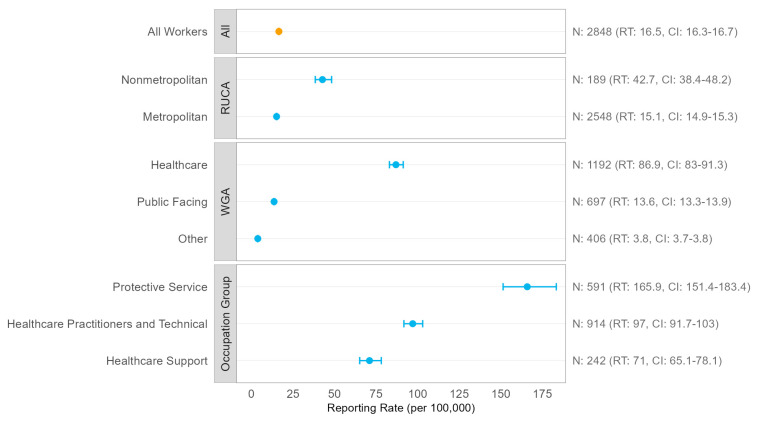
Reporting rates (RTs) for all California COVID-19 Doctor’s First Reports of Occupational Injury or Illness and with 2020–2021 average employment, stratified by overall, rural–urban commuting area (RUCA), worker group assignment (WGA), and occupation group (three most frequent occupation groups shown). The estimate in orange is for all workers, whereas the estimates in blue are estimates stratified by RUCA, WGA, or occupation group.

The overall reporting rate was 16.5 per 100,000 workers (CI: 16.3–16.7) (Figure 2). According to the worker group assignment, workers in the healthcare group had the highest reporting rate (86.9 per 100,000 workers, CI: 83.0–91.3), followed by workers in the public-facing group (13.6 per 100,000, CI: 13.3–13.9); however, when broken down by major occupation group, Protective Service workers, which include “bailiffs, correctional officers, and jailers” (COC 3800) and “police and sheriff’s patrol officers” (COC 3850), who are considered public-facing, had the highest reporting rate of 165.9 per 100,000 workers (CI: 151.4–183.4). The reporting rate was over two times higher among workers living in nonmetropolitan (42.7 per 100,000, CI: 38.4–48.2) than metropolitan RUCAs (15.1 per 100,000, CI: 14.9–15.3). Reporting rates were generally lower when calculated using just 2020 DFRs and 2020 CPS employment estimates, but there were no qualitative differences in the results between 2020 and all years (Supplementary Figure S1). 

### 3.3. Reported Exposures

Although over 60% of DFRs did not mention a source of SARS-CoV-2 exposure, over a quarter suspected that exposure was from a patient (19%) or client/customer (8%), and 11% mentioned a coworker exposure (Table 2). By worker group assignment, coworker exposures were over three times more frequent among public-facing (n = 132, 19%) and other (n = 81, 20%) workers than healthcare (n = 66, 5.5%) worker groups. By major occupation group, coworker exposures were most common in non-healthcare occupations such as Production (n = 13, 46%) and Construction and Extraction (n = 9, 39%) (Supplementary Table S1). Regarding the exposure site, a majority of DFRs were associated with workers in institutions (79%) and commercial, non-manufacturing sites (3%) (Table 2). There were no major differences between metropolitan and nonmetropolitan DFRs, except that nonmetropolitan (n = 5, 2.7%) DFRs had a higher proportion of agricultural sites compared with metropolitan DFRs (n = 17, <1%).

Overall, hospitals (56%) and prisons (18%) were the most common exposure sites. The use of PPE was unknown on nearly all DFRs (98%); 43 (1.5%) DFRs indicated that some amount of PPE was worn. 

Over two-thirds of all DFRs were generated from doctor office visits (36%) and telemedicine visits (35%), followed by emergency department visits (19%) (Table 2). There were differences in care-seeking behaviors of metropolitan and nonmetropolitan workers. Notably, doctor office visits were almost twice as frequent among nonmetropolitan workers (n = 118, 62%) compared with metropolitan workers (n = 858, 34%). Telemedicine visits were more common among metropolitan workers (n = 916, 36%) compared with nonmetropolitan workers (n = 39, 21%).

### 3.4. Reported Outcomes

Symptoms were noted on 68% of DFRs and were slightly more common among public-facing workers (n = 516, 74%) (Table 2). Overall, respiratory symptoms (52%) were the most frequently reported, followed by nervous system symptoms (40%), and constitutional/other (38%), among which fever (22%) and fatigue (18%) were most common (Supplementary Table S2). Among respiratory symptoms, cough (32%), dyspnea (18%), and sore throat (15%) were most common. Among nervous system symptoms, myalgias (23%) were most common, followed by headache (21%); anosmia or ageusia were noted on 12% of DFRs (Supplementary Table S2). 

**Figure 3:**
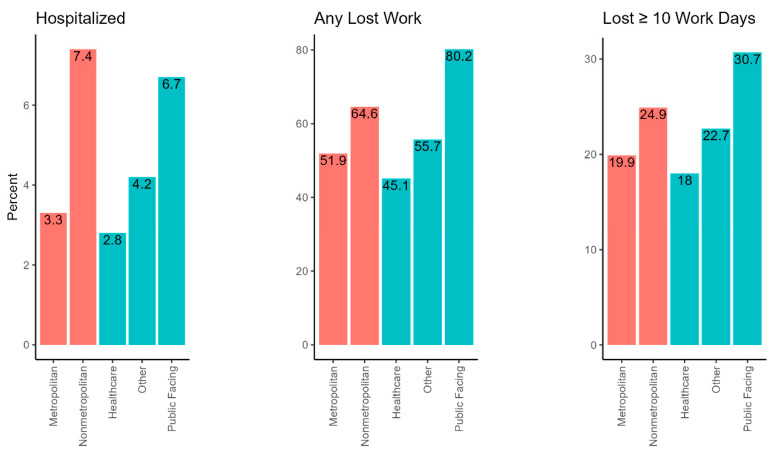
Comparing percent hospitalized, any lost work, and lost ≥ 10 days of work outcomes for COVID-19-related Doctor’s First Reports of Injury or Illness in California January 2020–February 2022, by rural–urban commuting area (RUCA) and worker group assignment (WGA). RUCA assignments are designated in red, and WGAs are designated in teal.

About half (54%) of all DFRs indicated some lost work time; compared with all DFRs, we found a greater proportion of lost work among nonmetropolitan (n = 122, 65%) and public-facing (n = 559, 80%) DFRs (Table 2). Among DFRs indicating lost time, nearly all (n = 1365/1525, 89.5%) lost time due to illness or symptoms. Notably, public-facing workers were more likely to lose ten or more workdays (n = 214, 31%) compared with all workers (21%), and especially when compared with healthcare workers (n = 215, 18%), who were least likely to lose ten or more workdays (Figure 3). Among major occupation groups with ten or more DFRs, Transportation and Material Moving (n = 17, 89.5%), Construction and Extraction (n = 20, 87%), and Protective Service (n = 480, 81%) had the highest proportion of workers with lost work (Supplementary Table S1). 

About 4% (n = 110) of DFRs indicated hospitalization for COVID-19 (Table 2). The proportion of DFRs indicating hospitalizations was higher among nonmetropolitan (n = 14, 7%) and public-facing (n = 47, 7%) workers compared with all DFRs. Among major occupation groups with ten or more DFRs, Transportation and Material Moving and Food Preparation and Serving Related occupations had the highest proportion of DFRs with hospitalizations. 

## 4. Discussion

DFRs for SARS-CoV-2 exposures and COVID-19 were concentrated in relatively few worksites and occupation groups, particularly in Healthcare Practitioners and Technical and Protective Service. Workers in healthcare (compared with public-facing occupations and other occupations) and those residing in nonmetropolitan RUCAs (compared with metropolitan) had a higher DFR reporting rate. DFRs from workers in public-facing occupations and those in nonmetropolitan areas had a higher-than-average proportion of hospitalizations and lost work time due to illness, suggesting more severe outcomes among these workers. 

While our findings are consistent with prior studies indicating that healthcare and protective service occupations have a higher risk for occupational COVID-19 compared with the general workforce [4,5,14], our results differ in important ways. For example, while over 300 COVID-19 outbreaks and thousands of outbreak-associated cases have been reported in California’s transportation industry, less than 20 DFRs were generated for workers in Transportation and Material Moving occupations [28]. Similarly, thousands of outbreak-associated cases have been reported in Accommodation and Food Services, Construction, and Manufacturing industries, but our DFRs yielded relatively few workers in these industries [5,6]. Given that thousands of workplace outbreaks have been reported and more than half of all DFRs we analyzed were from just five worksites, it is likely that most workers involved in outbreaks either do not seek care for exposures or illness or, when they do, physicians fail to recognize work-relatedness or fail to report [29]. 

Reporting physicians familiar with California’s Workers’ Compensation system and the “frontline worker” legal presumption for work-related COVID-19 may be more likely to submit DFRs for patients in those occupations (i.e., healthcare and protective services) than noncovered occupations [30]. Quigley et al.’s analysis of COVID-19 workers’ compensation claims in California mirrored our results in that workers in protective service and healthcare occupations had the highest claim rates, suggesting that legal presumptions which influence claim filings may also influence physician decisions to file DFRs [30]. Occupational culture may also influence care-seeking behaviors and DFR reporting. For example, it is common for workers in protective service and healthcare occupations to seek care and file workers’ compensation claims to formally document COVID-19 exposures, and this may be uncommon in other occupations [30]. 

While recent COVID-19 incidences have been higher in metropolitan counties, the cumulative and 7-day mortality rates remain higher in nonmetropolitan counties, suggesting a greater risk for severe COVID-19 outcomes in rural populations [16,31]. We see a similar pattern where the proportion of hospitalizations was two-times higher among nonmetropolitan than metropolitan DFRs. Furthermore, we estimated a higher proportion of lost work and higher DFR reporting rate among nonmetropolitan than metropolitan workers, suggesting that nonmetropolitan workers have both a greater risk for work-related COVID-19 and for severe outcomes. Our estimates of lost work may also be conservative given the high proportion of unknowns; the proportion of lost work is over 85% among nonmetropolitan DFRs with ascertainable lost work status. The higher rates of severe COVID-19 outcomes in rural populations may be due to lower COVID-19 vaccination coverage and further exacerbated by higher rates of comorbidities in these communities [18,32,33,34]. Further contributing to severe outcomes may be barriers to accessing care in rural communities, where there may be insufficient providers to meet the demand and geographic distances may delay care [17,19]. Notably, telehealth services were less often used among nonmetropolitan workers in our analysis but may be a means for expanding access to care for rural communities if known infrastructure barriers can be overcome [17,35]. 

DFRs from public-facing occupations—among which, the majority was in Protective Service occupations—had the highest proportion of hospitalizations and lost work, suggesting that they may be at risk for more severe COVID-19 outcomes. It is possible that these occupations may be less likely to report less severe disease. However, COVID-19 mortality data in California showed that Protective Service workers had a higher age-adjusted mortality rate compared to all other workers, thus further supporting our results [9]. Moreover, we found that Protective Service workers had the highest DFR reporting rate, suggesting that these workers have a high risk for work-related COVID-19 exposures. In their survey of 6000 Michigan workers taken in late 2020 through early 2021, Laskaris et al. found that Protective Service workers had the highest prevalence of reported workplace exposures of all occupation groups, notably even higher than Healthcare Support and Healthcare Practitioners and Technical occupations [36]. In nonpatient care settings, coworker exposures have been identified as a common transmission route [37]. We similarly found that the proportion of coworker exposures was highest in public-facing and other worker groups, suggesting that these exposures may be driving transmission in these occupations. 

This study had several limitations, including reporting bias, resulting in biased estimates of work-related SARS-CoV-2 exposures and COVID-19. DFRs likely do not represent all workers affected by occupational COVID-19 given the prolonged reporting chain [29]. Reporting requires workers to seek medical care; the treating physician then must recognize work-relatedness and know about reporting requirements and complete a DFR. Furthermore, concerns of workplace retribution deter workers from reporting. Prior studies have indicated that up to two-thirds of occupational injuries and illnesses are not captured in DFRs, suggesting that a large proportion of occupational COVID-19 may be unreported [21]. It is possible that severe disease was more likely to be reported due to lost work time and need for compensation; this may differ by region or occupation and could account for some of the associations observed. Moreover, nearly a fifth of the DFRs analyzed lacked sufficient detail to code worker occupation and likely resulted in an underestimate of DFRs in certain occupation groups. DFRs may not be useful for assessing PPE use during a pandemic; nearly all DFRs lacked any detail about PPE use. Our estimate of lost work time may be inaccurate since the counts of lost workdays were based on an assumed standard business work schedule and over a third of DFRs lacked lost work details. Finally, SARS-CoV-2 exposure is often unknown, and the exposure source was missing for over half of the DFRs analyzed. The exposure source and case-site details were based on DFR narratives, which depended on patients suspecting how and where they were exposed, reporting that exposure to physicians, and the physician then recording it on the DFR. 

Despite limitations, our findings have implications for public health. First, while DFRs are an important source of occupational COVID-19 surveillance and capture rich details about COVID-19 near the time of illness, DFRS are not necessarily representative of the California workforce. A systematic collection of industry and occupation for all COVID-19 cases would greatly enhance our understanding of occupational COVID-19. Second, interventions to reduce severe COVID-19 outcomes in nonmetropolitan workers are needed, such as increasing COVID-19 vaccine acceptance and uptake; the CDC has published guidance on increasing vaccine confidence and access in rural communities that health departments could implement [38]. Finally, given their high reporting rate, public-facing employers can implement workplace mitigation measures to reduce transmission [39]. Presently, employers can help employees get vaccinated (e.g., providing paid time off to do so), instruct exposed workers to get tested, provide and encourage the use of paid sick leave, provide and support the use of masks, maintain ventilation systems, conduct routine disinfection, and confer with OSHA recommendations to protect workers from COVID-19 [39]. 

## 5. Conclusions

Healthcare and prison workers were the majority of cases of SARS-CoV-2 exposure and COVID-19 illnesses in the DFR surveillance system. However, their overrepresentation in the DFR surveillance data may not be representative of SARS-CoV-2 exposure and COVID-19 illness among California workers overall. The incomplete surveillance of occupational COVID-19 has hindered public health response to identify and protect vulnerable workers. Additional outreach to physicians to educate them about recognizing work-related COVID-19 may improve reporting. Moreover, accurate reporting of industry and occupation in other data sources would enhance our understanding of occupational COVID-19. DFR surveillance differs from other surveillance systems, in that industry and occupation data are relatively complete and available. DFR surveillance helped address this knowledge gap and provided additional insights into workers who may need further public health support. Public health surveillance systems should require the collection of industry and occupation data. Future work to enhance occupational health surveillance will be needed to ensure that accurate, timely, and actionable data will be available in future outbreaks. 

## Figures and Tables

**Table 1:** Workers whose SARS-CoV-2 exposures or COVID-19 diagnoses were reported by physicians to the state of California via Doctor’s First Report of injury or Illness (DFR), stratified by rural–urban commuting area (RUCA) and worker group assignment—January 2020–February 2022.

	All Workers N (%)	Rural–Urban Commuting Area		Worker Group Assignment		
		Metropolitan N (%)	Nonmetropolitan N (%)	Public Facing N (%)	Healthcare N (%)	Other N (%)
**N**	2848	2548	189	697	1192	406
**Age, median (IQR)**	42 (32, 51)	42 (32, 51)	42 (32, 51.25)	42 (33, 49)	40.5 (32, 53)	44 (35, 54)
**Sex**						
Male	1281 (44.98)	1103 (43.29)	125 (66.14)	507 (72.74)	335 (28.1)	177 (43.6)
Female	1453 (51.02)	1340 (52.59)	63 (33.33)	190 (27.26)	848 (71.14)	228 (56.16)
Unknown	114 (4)	ꝉ	ꝉ	–	ꝉ	ꝉ
**Rural–Urban Commuting Area**						
Metropolitan	2548 (89.47)	2548 (100)	–	566 (81.21)	1125 (94.38)	376 (92.61)
Nonmetropolitan	189 (6.64)	–	189 (100)	86 (12.34)	32 (2.68)	22 (5.42)
Unknown	111 (3.9)	–	–	45 (6.46)	35 (2.94)	8 (1.97)
**Worker Group Assignment**						
Public Facing Occupation	697 (24.47)	566 (22.21)	86 (45.5)	697 (100)	–	–
Healthcare Occupation	1192 (41.85)	1125 (44.15)	32 (16.93)	–	1192 (100)	–
Other Occupations	406 (14.26)	376 (14.76)	22 (11.64)	–	–	406 (100)
Unknown	553 (19.42)	481 (18.88)	49 (25.93)	–	–	–
Occupation Group *						
Architecture and Engineering	6 (0.21)	6 (0.24)	–	–	–	6 (1.48)
Building and Grounds Cleaning and Maint.	55 (1.93)	ꝉ	ꝉ	–	–	55 (13.55)
Business and Financial Operations	17 (0.6)	ꝉ	ꝉ	ꝉ	–	ꝉ
Community and Social Service	25 (0.88)	ꝉ	ꝉ	25 (3.59)	–	–
Construction and Extraction	23 (0.81)	ꝉ	ꝉ	–	–	23 (5.67)
Education, Training, and Library	20 (0.7)	14 (0.55)	5 (2.65)	20 (2.87)	–	–
Farming, Fishing, and Forestry	23 (0.81)	17 (0.67)	5 (2.65)	–	–	23 (5.67)
Food Preparation and Serving Related	31 (1.09)	23 (0.9)	6 (3.17)	18 (2.58)	–	13 (3.2)
Healthcare Practitioners and Technical	914 (32.09)	864 (33.91)	23 (12.17)	–	914 (76.68)	–
Healthcare Support	242 (8.5)	226 (8.87)	8 (4.23)	–	242 (20.3)	–
Installation, Maintenance, and Repair	30 (1.05)	ꝉ	ꝉ	–	–	30 (7.39)
Management	56 (1.97)	ꝉ	ꝉ	5 (0.72)	36 (3.02)	15 (3.69)
Office and Administrative Support	189 (6.64)	175 (6.87)	10 (5.29)	17 (2.44)	–	172 (42.36)
Personal Care and Service	10 (0.35)	10 (0.39)	–	10 (1.43)	–	–
Production	28 (0.98)	ꝉ	ꝉ	–	–	28 (6.9)
Protective Service	591 (20.75)	482 (18.92)	69 (36.51)	588 (84.36)	–	ꝉ
Sales and Related	9 (0.32)	ꝉ	ꝉ	6 (0.86)	–	ꝉ
Transportation and Material Moving	19 (0.67)	ꝉ	ꝉ	ꝉ	–	15 (3.69)
Unknown	552 (19.38)	480 (18.84)	49 (25.93)	–	–	–
Industry Group *						
Accommodation and Food Services	17 (0.6)	ꝉ	ꝉ	ꝉ	–	8 (1.97)
Administrative and Support and Waste Mgmt.	33 (1.16)	33 (1.3)	–	ꝉ	ꝉ	12 (2.96)
Agriculture, Forestry, Fishing, and Hunting	83 (2.91)	62 (2.43)	18 (9.52)	30 (4.3)	–	28 (6.9)
Construction	8 (0.28)	ꝉ	–	–	–	ꝉ
Educational Services	42 (1.47)	28 (1.1)	11 (5.82)	27 (3.87)	–	11 (2.71)
Healthcare and Social Assistance	1693 (59.45)	1606 (63.03)	43 (22.75)	55 (7.89)	1141 (95.72)	203 (50)
Manufacturing	16 (0.56)	ꝉ	ꝉ	–	–	5 (1.23)
Other Services, Except Public Administration	7 (0.25)	7 (0.27)	–	–	–	ꝉ
Public Administration	778 (27.32)	642 (25.2)	90 (47.62)	558 (80.06)	45 (3.78)	60 (14.78)
Real Estate and Rental and Leasing	16 (0.56)	16 (0.63)	–	–	–	ꝉ
Retail Trade	20 (0.7)	ꝉ	ꝉ	5 (0.72)	–	9 (2.22)
Transportation and Warehousing	25 (0.88)	ꝉ	ꝉ	ꝉ	–	15 (3.69)
Utilities	49 (1.72)	ꝉ	ꝉ	–	ꝉ	37 (9.11)
Unknown	44 (1.54)	18 (0.71)	15 (7.94)	9 (1.29)	ꝉ	ꝉ

* Occupations (Arts, Design, Entertainment, Sports, and Media; Computer and Math; Life, Physical and Social Science; Military) and industries (Arts, Entertainment, and Recreation; Finance and Insurance; Information; Management of Companies and Enterprises; Professional, Scientific, and Technical Services; Wholesale Trade) with less than 5 DFRs not shown. ꝉ Cells with less than 5 DFRs (and complementary cells) within a given category are suppressed. Cells with 0 DFRs (NA) are displayed as “–”.

**Table 2:** SARS-CoV-2 exposure and COVID-19 outcome details of workers reported via Doctor’s First Report of injury or Illness (DFR) in California, stratified by rural–urban commuting area (RUCA) and worker group assignment (WGA), January 2020–February 2022.

		Rural–Urban Commuting Area		Worker Group Assignment		
	All Workers N (%)	Metropolitan N (%)	Nonmetropolitan N (%)	Public Facing N (%)	Healthcare N (%)	Other N (%)
**N**	2848	2548	189	697	1192	406
**Exposures**						
**Suspected Source**						
Patient	540 (18.96)	516 (20.25)	11 (5.82)	36 (5.16)	427 (35.82)	51 (12.56)
Client/Customer	240 (8.43)	208 (8.16)	10 (5.29)	152 (21.81)	63 (5.29)	14 (3.45)
Coworker	324 (11.38)	284 (11.15)	29 (15.34)	132 (18.94)	66 (5.54)	81 (19.95)
Personal	50 (1.76)	46 (1.81)	ꝉ	8 (1.15)	24 (2.01)	11 (2.71)
Not Stated	1739 (61.06)	1534 (60.2)	139 (73.54)	400 (57.39)	627 (52.6)	247 (60.84)
Unknown to Patient	46 (1.62)	43 (1.69)	ꝉ	10 (1.43)	18 (1.51)	12 (2.96)
Exposure Site/Worksite *						
Institution	2249 (78.97)	2040 (80.06)	121 (64.02)	459 (65.85)	1177 (98.74)	239 (58.87)
Commercial Facility, Non-Mfg.	90 (3.16)	83 (3.26)	5 (2.65)	13 (1.87)	–	52 (12.81)
Other	56 (1.97)	46 (1.81)	8 (4.23)	30 (4.3)	2 (0.17)	14 (3.45)
Agricultural	23 (0.81)	17 (0.67)	5 (2.65)	2 (0.29)	–	14 (3.45)
Manufacturing	13 (0.46)	13 (0.51)	–	1 (0.14)	–	6 (1.48)
More than One Site	5 (0.18)	ꝉ	ꝉ	2 (0.29)	3 (0.25)	–
Unknown	411 (14.43)	344 (13.5)	49 (25.93)	189 (27.12)	10 (0.84)	81 (19.95)
**Type of Care for DFR**						
Doctor’s Office	1036 (36.38)	858 (33.67)	118 (62.43)	333 (47.78)	326 (27.35)	135 (33.25)
Emergency Department	530 (18.61)	513 (20.13)	12 (6.35)	34 (4.88)	286 (23.99)	90 (22.17)
Hospital Admission	8 (0.28)	8 (0.31)	–	4 (0.57)	3 (0.25)	–
No Medical Care Sought	8 (0.28)	8 (0.31)	–	6 (0.86)	–	2 (0.49)
Employee Health Center	188 (6.6)	175 (6.87)	10 (5.29)	6 (0.86)	133 (11.16)	22 (5.42)
Telemedicine	995 (34.94)	916 (35.95)	39 (20.63)	297 (42.61)	423 (35.49)	141 (34.73)
Unknown	83 (2.91)	70 (2.75)	10 (5.29)	17 (2.44)	21 (1.76)	16 (3.94)
**Outcomes**						
**Any Symptoms**						
Yes	1932 (67.84)	1727 (67.78)	118 (62.43)	516 (74.03)	802 (67.28)	281 (69.21)
None Reported	916 (32.16)	821 (32.22)	71 (37.57)	181 (25.97)	390 (32.72)	125 (30.79)
Hospitalized **						
Yes	110 (3.86)	85 (3.34)	14 (7.41)	47 (6.74)	33 (2.77)	17 (4.19)
No	2738 (96.14)	2463 (96.66)	175 (92.59)	650 (93.26)	1159 (97.23)	389 (95.81)
**Lost Work**						
Yes	1525 (53.55)	1322 (51.88)	122 (64.55)	559 (80.2)	538 (45.13)	226 (55.67)
No	338 (11.87)	312 (12.24)	17 (8.99)	38 (5.45)	226 (18.96)	62 (15.27)
Unknown	985 (34.59)	914 (35.87)	50 (26.46)	100 (14.35)	428 (35.91)	118 (29.06)
**Lost Work Reason**						
Due to Illness/Symptoms	1365 (47.93)	1181 (46.35)	109 (57.67)	491 (70.44)	469 (39.35)	209 (51.48)
Quarantine or Asymptomatic ***	160 (5.62)	141 (5.53)	13 (6.88)	68 (9.76)	69 (5.79)	17 (4.19)
No Lost Time	338 (11.87)	312 (12.24)	17 (8.99)	38 (5.45)	226 (18.96)	62 (15.27)
Unknown	985 (34.59)	914 (35.87)	50 (26.46)	100 (14.35)	428 (35.91)	118 (29.06)
Lost Time Amount ****						
<10 days	472 (16.57)	418 (16.41)	29 (15.34)	219 (31.42)	139 (11.66)	56 (13.79)
≥10 days	592 (20.79)	507 (19.9)	47 (24.87)	214 (30.7)	215 (18.04)	92 (22.66)
Lost Work but Unknown Amount	461 (16.19)	397 (15.58)	46 (24.34)	126 (18.08)	184 (15.44)	78 (19.21)
Unknown	985 (34.59)	914 (35.87)	50 (26.46)	100 (14.35)	428 (35.91)	118 (29.06)

* One report of exposure in private residence (not shown). ** Includes hospitalizations that preceded the visit for the DFR in the course of the illness. *** Asymptomatic with positive COVID-19 test result. **** Excludes those who did not lose work time. ꝉ Cells with less than 5 DFRs within a given RUCA category are not shown. Cells with 0 DFRs (NA) are displayed as “–”.

## Data Availability

The data presented in this study are not available due to privacy restrictions.

## Supplementary Materials

The following supporting information can be downloaded at https://www.mdpi.com/article/10.3390/ijerph20136307/s1, Table S1: Demographics, exposures, and outcomes among represented among Doctor’s First Report of Occupational Injury or Illness (DFR) included in analysis stratified by occupation major group, California, January 2020–February 2022. Table S2: Frequency of detailed symptoms by body system represented among Doctor’s First Report of Occupational Injury or Illness (DFR), California, January 2020–February 2022. Figure S1: Reporting rates for California 2020 DFRs and with 2020 average employment, stratified by overall, rural–urban commuting area (RUCA), worker group assignment (WGA), and occupation group. Figure S2: Reporting rates for all California DFRs and with 2020–2021 average employment, stratified by overall, rural–urban commuting area (RUCA), worker group assignment (WGA), and occupation group. Description of algorithm and keywords used to screen for COVID-19 DFRs.
