# Supplementary material for: Occupational COVID-19 Exposures and Illnesses among Workers in California—Analysis of a New Occupational COVID-19 Surveillance System
Source: Int J Environ Res Public Health. 2023 Jul 6;20(13):6307. doi: 10.3390/ijerph20136307 (PMC10341532; doi:10.3390/ijerph20136307)
Supplement: Supplementary file 1 [file ijerph-20-06307-s001.zip › 7. OHB DFR Manuscript Supplemental materials.pdf]

DFR Manuscript Supplemental Materials

1. **Description of algorithm and keywords used to screen for COVID-19 DFRs**

    1. **Screen DFR for COVID-19 diagnosis.**
        a. If missing or other diagnosis is listed (for example asthma, suspected/exposure to covid, contact w and exposure to other communicable diseases), look for positive COVID-19 test result and a mention of COVID-19 anywhere in the DFR.
    2. **Screen for COVID-19 associated respiratory signs and symptoms.**
        a. Cough, shortness of breath, wheeze, chest tightness
    3. **Screen for other COVID-19 associated symptoms.**
        a. Fever, runny nose, congestion, diarrhea, headache, body ache, loss of sense of smell/taste, chills, fatigue.
    4. **Search for mention of work-relatedness.**
        a. "Contracted covid at work" or "exposed to coworker who tested positive" or "worked with clients/patients with covid"
    5. **Search for mention of "vaccination" and/or "booster" status**
    6. **Screen for history of prior COVID-19 exposure or illness.**
        a. Long haul covid
        b. Past medical history of covid
        c. Mention of first time or second time with covid

2. **State of California Doctor's First Report Form 5021.**

5021.pdf (ca.gov)
