# Supplementary figures and images for: Occupational COVID-19 Exposures and Illnesses among Workers in California—Analysis of a New Occupational COVID-19 Surveillance System
Source: Int J Environ Res Public Health. 2023 Jul 6;20(13):6307. doi: 10.3390/ijerph20136307 (PMC10341532; doi:10.3390/ijerph20136307)

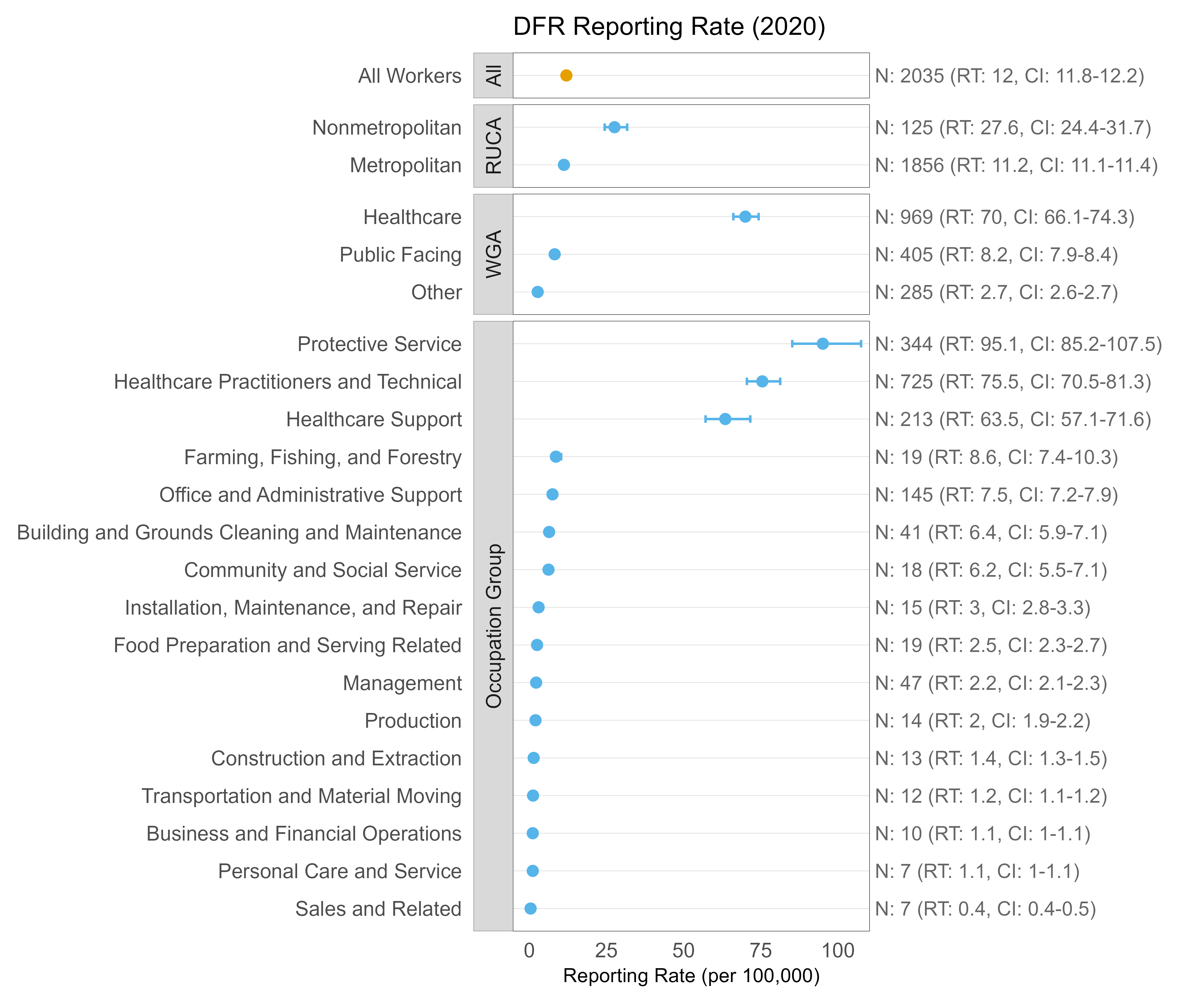

Supplement: Supplementary file 1 [file ijerph-20-06307-s001.zip › Supplement Figure S1.TIFF]
